# NIH funding in the biophotonics community

**DOI:** 10.1117/1.BIOS.3.2.020601

**Published:** 2026-06-05

**Authors:** Aarohi Mahesh Mehendale, Darren Roblyer

**Affiliations:** a Boston University, Department of Biomedical Engineering, Boston, Massachusetts, United States; b Boston University, Department of Electrical and Computer Engineering, Boston, Massachusetts, United States

**Keywords:** funding, biophotonics community, NIH, institutes, strengths, opportunities

## Abstract

**Significance:**

This work provides a data-driven characterization of NIH funding to the biophotonics community, offering investigators, researchers, and funding agencies a clearer picture of where the field excels and where opportunities for growth remain.

**Aim:**

We aim to characterize the current NIH funding landscape supporting core biophotonics technologies and identify strengths, gaps, and opportunities for future growth.

**Approach:**

NIH-funded biophotonics projects in 2024 were identified using two complementary strategies: (1) a broad keyword-driven search of NIH RePORTER and (2) a targeted investigator-centered search based on participation in biophotonics-focused journals and conferences. Project abstracts were analyzed using a large language model to perform keyword clustering and automated classification across multiple dimensions, including funding institute, optical modality, clinical/disease focus, and geographic distribution. A focused analysis of the National Institute of Biomedical Imaging and Bioengineering (NIBIB) portfolio was also performed.

**Results:**

The broad query captured ∼$3.3 billion in 2024 grant funding that may be connected to biophotonics. However, optical technologies were peripheral to the primary research aims in a substantial fraction of these awards. The targeted community-based approach identified $288 million in funding directly aligned with core biophotonic imaging and sensing. Cancer, neuroscience, and vision collectively represented 44% of this direct portfolio. The most common modalities included microscopy, endoscopy, fluorescence/bioluminescence imaging, and optical coherence tomography. Funding related to chronic diseases (e.g., cardiovascular and kidney disease), infectious diseases, aging, and reproductive and child health was proportionally underrepresented relative to NIH-wide priorities.

**Conclusions:**

NIH exhibits strong support for biophotonics innovation in cancer, neuroscience, and vision science, reflecting historical strengths of the field. However, a substantial opportunity exists to expand impact toward areas of high national health burden where optical technologies remain underutilized. These data may help inform NIH institute-level strategies and community advocacy efforts to align future biophotonics investments with broader public health needs.

Statement of Discovery This work systematically maps the NIH biophotonics funding landscape, clarifying which institutes drive current investment and how the supported research areas align, or misalign, with broader NIH health priorities. The findings highlight both established strengths and strategic opportunities for expanded impact.

## 1 Introduction

The National Institutes of Health (NIH) is the world’s largest public supporter of biomedical research, providing more than one-third of global investment in life sciences discovery and translation.^1^ Across its 27 institutes and centers, NIH supports fundamental science, clinical research, workforce development, and small business innovation that align with major national initiatives. These priorities include the Cancer Moonshot,^2^ the BRAIN Initiative for neuroscience,^3^ the *All of Us* Research Program for precision health,^4^ the HEAL Initiative focused on pain and opioid misuse,^5^ and renewed focus on aging, infectious diseases, maternal and child health, and health equity.

Biophotonics resides at a critical interface between engineering innovation and biomedical impact. Optical imaging, spectroscopy, and sensing technologies enable minimally invasive, high-resolution, and often portable approaches to disease detection, therapeutic monitoring, and mechanistic discovery. Although the field receives significant NIH investment across multiple institutes, the specific distribution of this support by institute, disease area, optical modality, and national geography remains poorly defined.

Rapid and substantial changes are occurring across the NIH portfolio, including evolving strategic priorities, new funding mechanisms, and significant organizational shifts. For the biophotonics community to continue advancing translational impact, it is essential to understand where our field currently stands within this changing landscape and where strategic adaptation may be needed.

Here, we analyze NIH support for biophotonics research in 2024 using a combined data-driven and community-anchored approach, providing insight into current strengths and opportunities for future growth. We anticipate that this analysis can inform both investigators and decision makers at NIH and other funding organizations seeking to align investments with areas of emerging opportunity for optical technologies in medicine.

## 2 Current Funding Landscape in Biophotonics

**Fig. 1:**
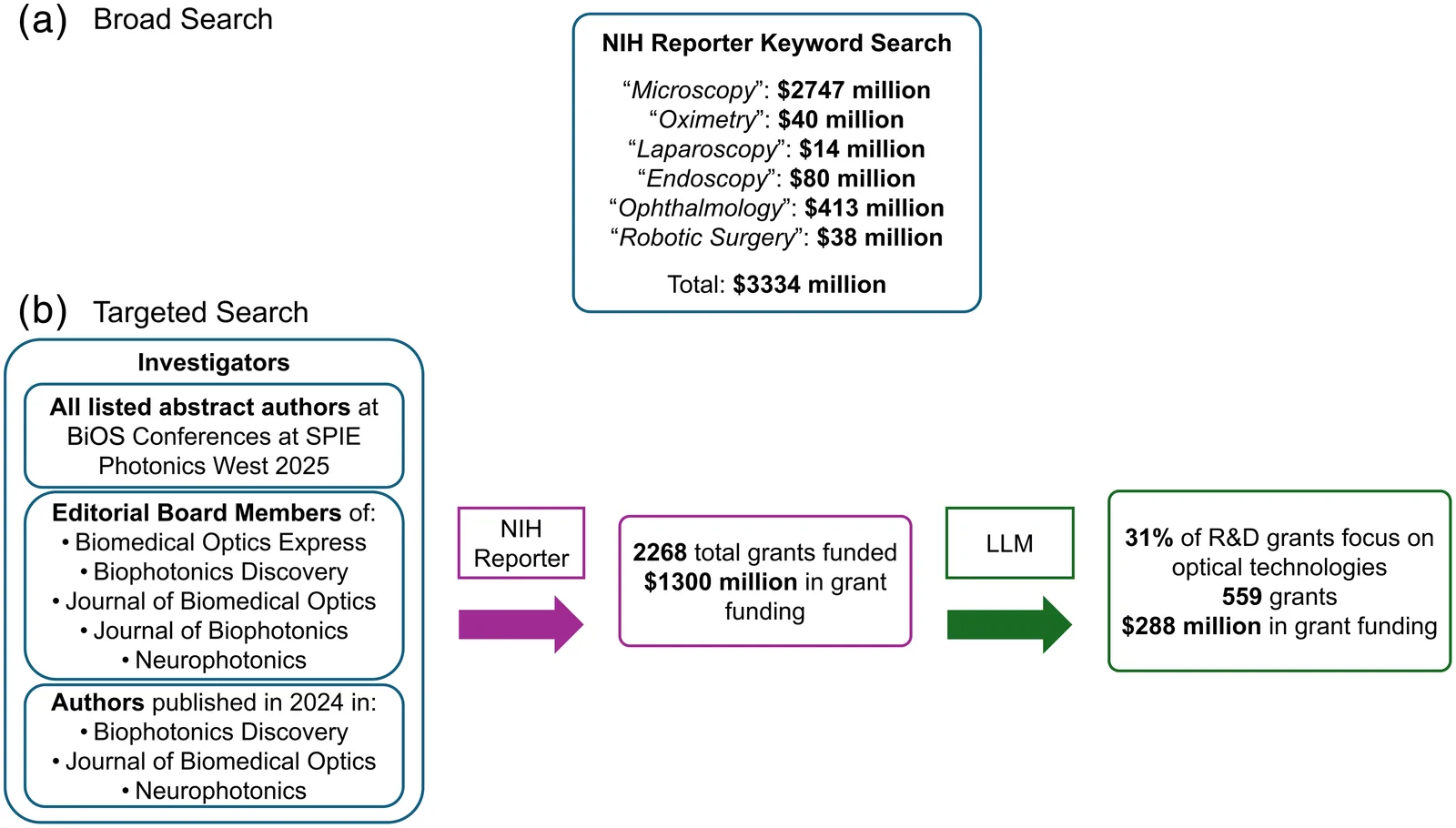
Overview of the data collection strategy. A broad keyword search in NIH RePORTER provided an upper bound on NIH funding related to biophotonics in 2024. A more targeted investigator-based approach identified NIH-funded principal investigators active in core biophotonics venues. Research and development grants were then analyzed using a large language model to determine which projects primarily involve optical technologies.

To understand the current placement of biophotonics within NIH priorities, we examined fiscal year (FY) 2024 NIH funding using two complementary data collection strategies (Fig. 1). This included all grants active in FY 2024, irrespective of when the grants were awarded. A broad keyword search in NIH RePORTER^6^ identified ∼$3334 million in projects connected to optical technologies. This provides an upper bound on NIH investment related to imaging and sensing, and aligns with past analysis^7^ but optical tools were often peripheral to project goals, with many of these projects simply using optical tools such as microscopy as part of their analysis toolset.

To more precisely capture core biophotonics activities, we constructed a field-anchored investigator list based on participation in major biophotonics journals and the SPIE Photonics West conference. Inclusion required at least one of the following: attendance at SPIE Photonics West 2025 (data provided by SPIE), editorial board membership on *Biophotonics Discovery*,^8^ *Journal of Biomedical Optics*,^9^ *Neurophotonics*,^10^ *Journal of Biophotonics*,^11^ or *Biomedical Optics Express*,^12^ or authorship contributions to *Biophotonics Discovery*, *Journal of Biomedical Optics*, or *Neurophotonics* in 2024 or 2025 (data provided by SPIE) (Fig. 1). This approach ensures a strong connection to active optical imaging, spectroscopy, and sensing research.

Grants linked to these investigators were extracted from NIH RePORTER and filtered to focus exclusively on research and development awards, as defined by the NIH’s funding categories.^13^ This does not include small business grants (STTR/SBIR) or individual or institutional training grants. The filtered project abstracts were then evaluated using a large language model (LLM) through the tidyllm package in R (OpenAI, GPT-4o,2024-08-06). The model performed keyword clustering and automated classification to identify the optical technology used and the primary clinical or biological application area. Additional validation and error checking were performed in ChatGPT5 (OpenAI, 2025), including manual inspection of any ambiguous cases. All prompts and decision rules are provided in Supplementary Material 2. The final dataset of investigators, grants, and annotations is also provided in the supplement for transparency and reproducibility.

The curated dataset identified 559 grants with a major focus on optical technologies, representing $288 million in FY 2024 support. These results show that while biophotonics contributes broadly across NIH research, the focused optical technologies community represents approximately one quarter of the investment captured by keyword searches. This narrowing illustrates the importance of a community-based approach when assessing the true biophotonics funding landscape.

## 3 Institute Support Patterns Reflect Historical Strengths

**Fig. 2:**
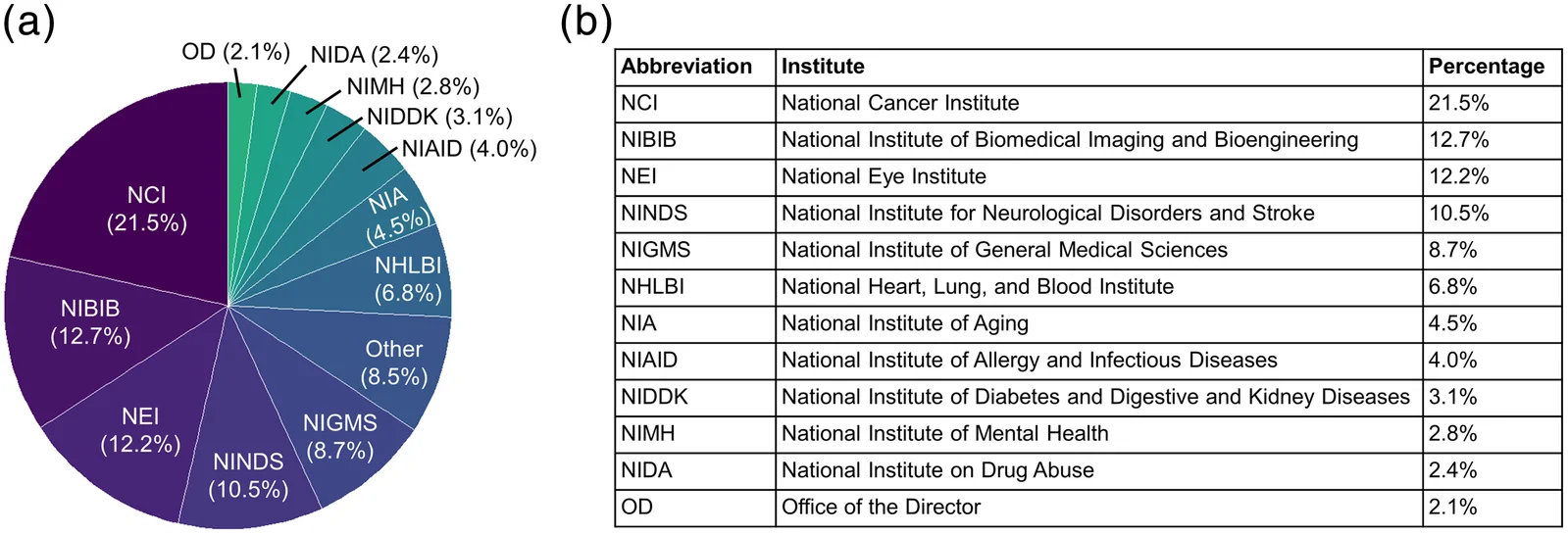
(a) Percentage of the total number of grants received by the community by funding institute. “Other” refers to institutes not listed that contribute less than 2% to the total funding received by the biophotonics community. (b) Names of the top 12 institutes that fund investigators in the biophotonics community.

A total of 24 NIH funding institutes supported biophotonics research in 2024, and those contributing more than 2% are highlighted (Fig. 2). The National Cancer Institute (NCI) funded the greatest number of grants in the biophotonics community at 123 (21.5% by number of grants, $54 million of total funding). This was 40% more in funding dollars than the next-ranked institute, which was the National Institute of Biomedical Imaging and Bioengineering (NIBIB) (73 grants, 12.7%, $29 million). These were followed by the National Eye Institute (NEI) (70 grants, 12.2%, $31 million), the National Institute of Neurological Disorders and Stroke (NINDS) (60 grants, 10.5%, $36 million), the National Institute of General Medical Sciences (NIGMS) (50 grants, 8.7%, $17 million), the National Heart, Lung, and Blood Institute (NHLBI) (39 grants, 6.8%, $23 million), and the National Institute on Aging (NIA) (26 grants, 4.5%, $18 million).

## 4 Strengths Relative to NIH-Wide Priorities

**Fig. 3:**
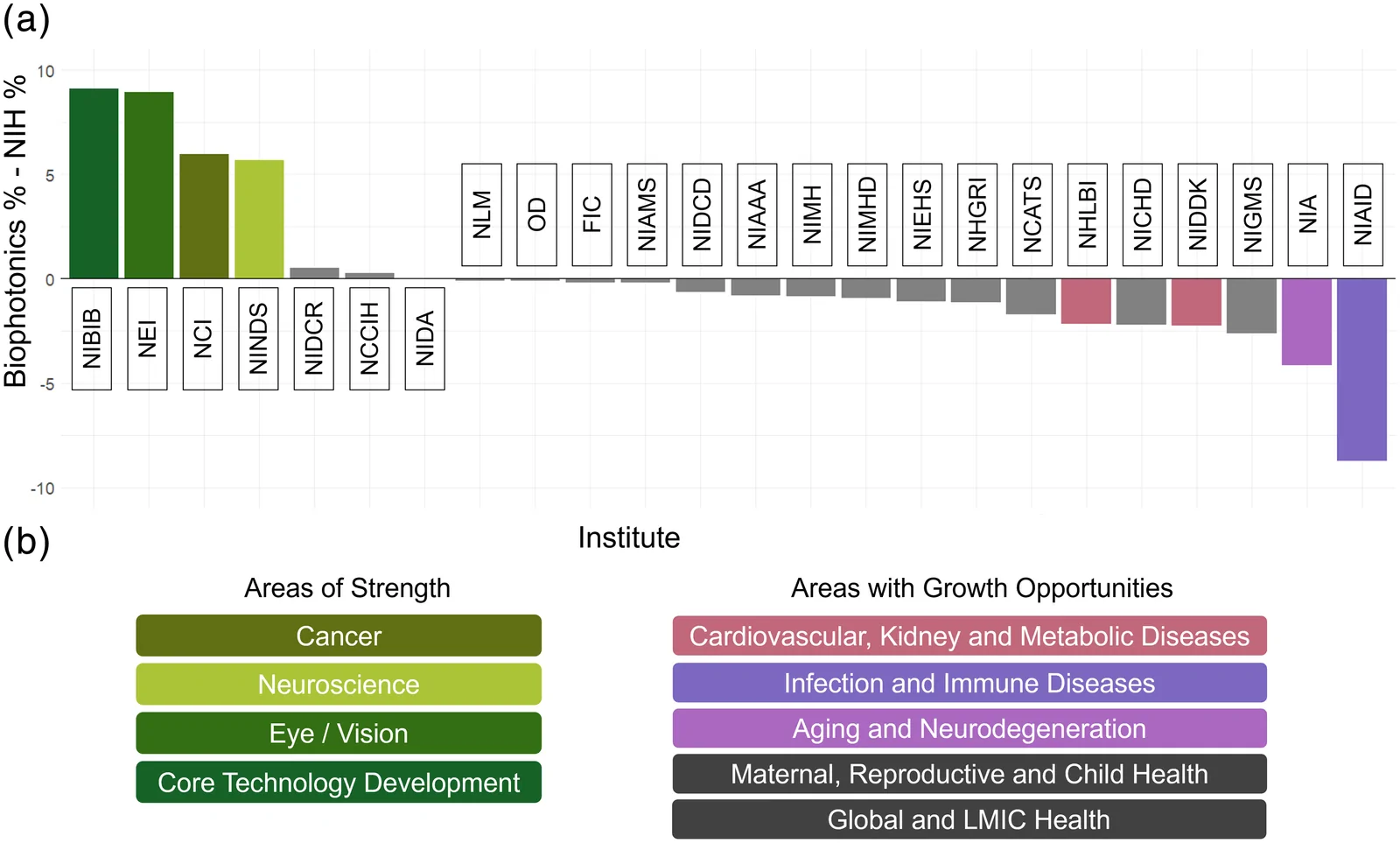
(a) Relative over- and under-representation of biophotonics funding by the NIH institute and office. Bars show the difference between the percentage of total biophotonics funding and the percentage of the overall NIH extramural portfolio for each institute (biophotonics percent minus NIH percent). Institutes with positive values on the y-axis represent “strengths,” where biophotonics receives a larger share of funding than expected based on the NIH portfolio, whereas institutes with negative values on the y-axis represent “opportunities,” where biophotonics is proportionally underrepresented. (b) Topic-level strengths and opportunities for NIH-funded biophotonics. NCCIH: National Center for Complementary and Integrative Health. NLM: National Library of Medicine. FIC: Fogarty International Center. NIAMS: National Institute of Arthritis and Musculoskeletal and Skin Diseases. NIDCD: National Institute on Deafness and Other Communication Disorders. NIAAA: National Institute on Alcohol Abuse and Alcoholism. NIMHD: National Institute on Minority Health and Health Disparities. NIEHS: National Institute of Environmental Health Sciences. NHGRI: National Human Genome Research Institute. NCATS: National Center for Advancing Translational Sciences. LMIC: Low- and middle-income country. Note: Fig. 2(b) identifies other NIH acronyms.

When compared with the overall NIH extramural funding portfolio, cancer and neuroscience stand out as particular strengths of the biophotonics field (Fig. 3). NCI and NINDS together represent more than 30% of biophotonics funding by dollar amount. NEI and NIBIB also support a disproportionately large share of optical technology development, consistent with the close alignment of imaging needs in ophthalmology and the central technology mission of NIBIB. These areas reflect mature and clinically embedded optical platforms, including OCT in ophthalmology and multimodality imaging in cancer and neuroscience.

## 5 Gaps and Growth Opportunities

By contrast, institutes focused on chronic disease, infection, aging, childhood development, and metabolic health show comparatively lower biophotonics engagement (Fig. 3). The largest gaps, relative to NIH spending priorities, are seen in the National Institute of Allergy and Infectious Diseases (NIAID), the National Institute of Diabetes and Digestive and Kidney Diseases (NIDDK), the National Institute of Child Health and Human Development (NICHD), NIA, NIGMS, and NHLBI.

A small fraction of funded biophotonics grants focus on global health (10.7%) or women’s, maternal, and neonatal health (7%), despite significant national and global health burdens in these areas. This points to opportunities for increased engagement with institutes and clinical partners working in these priority domains.

These gaps suggest substantial room for expansion into cardiovascular disease, infectious disease surveillance, immune dysfunction, diabetes, and maternal and child health.

## 6 Optical Modality Distribution

**Fig. 4:**
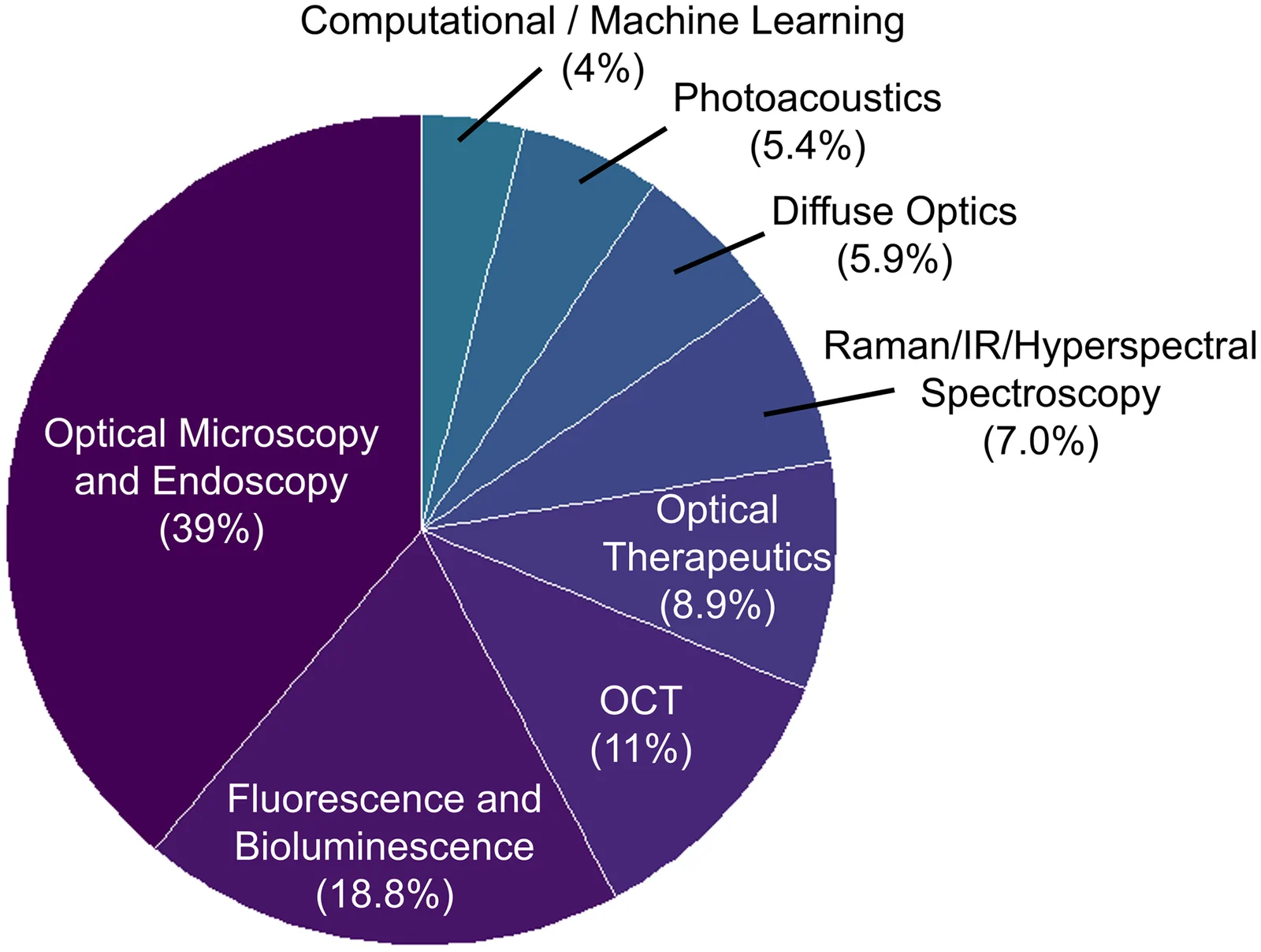
Percentage of grants by optical technology.

Large language model (LLM)-driven classification of abstracts shows that microscopy and endoscopy dominate current NIH-funded biophotonics work, comprising 39% of projects (Fig. 4). Fluorescence and bioluminescence imaging accounted for 18.8%, and OCT represented 11%. These data underscore the continued strength and widespread use of established imaging platforms within NIH-supported biophotonics research.

## 7 Geographic Distribution

**Fig. 5:**
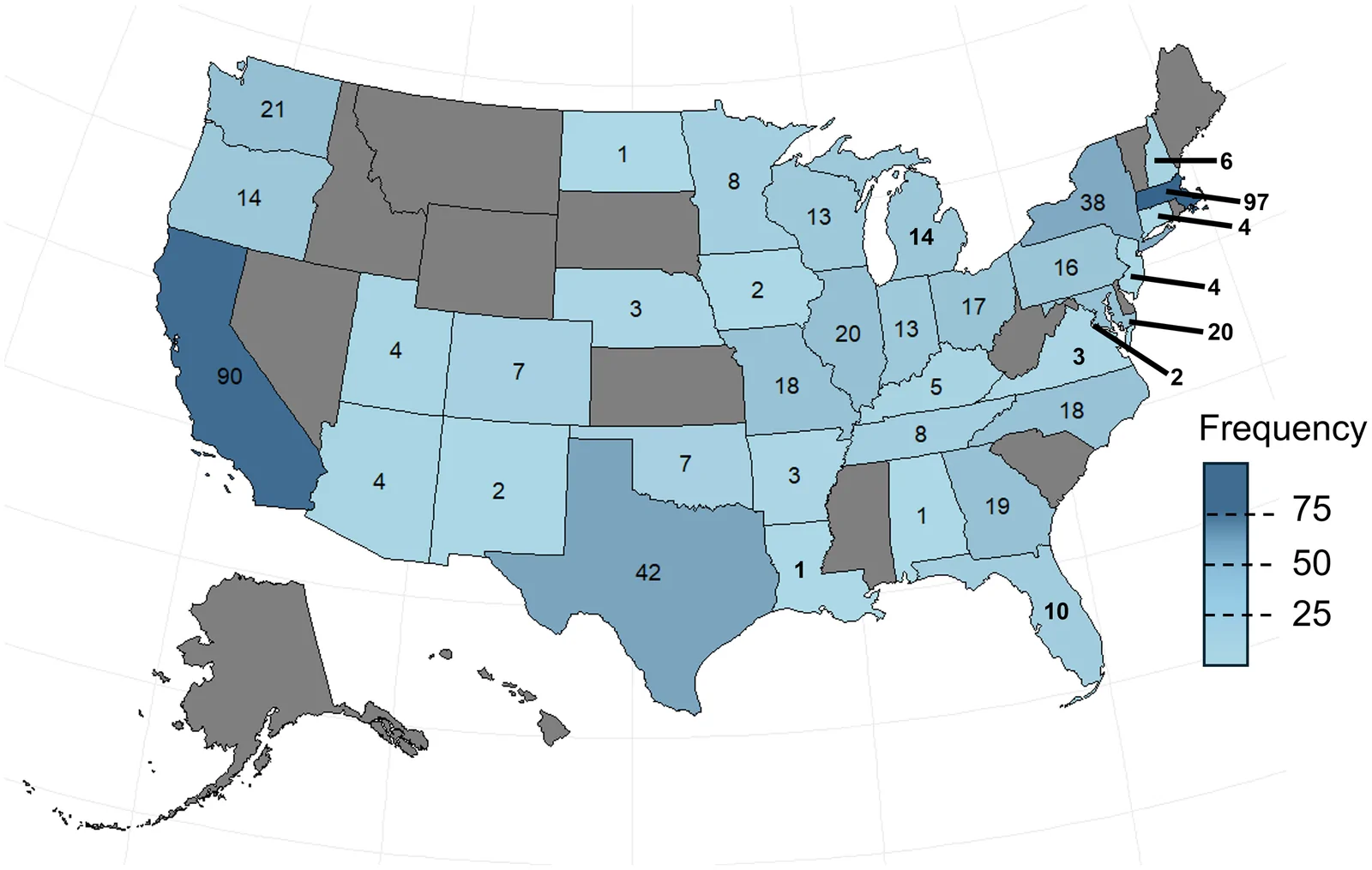
United States map, color-coded by the number of grants received by the institutions in the respective states. Darker shades represent a higher number of grants. Numbers within the states represent the number of grants.

Nearly all biophotonics funding in FY 2024 was awarded to institutions within the United States (99.8%). States with the highest number of grants included Massachusetts (97 grants), California (90 grants), Texas (42 grants), and New York (38 grants) (Fig. 5). These geographic concentrations reflect the strong presence of established optical imaging clusters and innovation ecosystems that have grown around them.

## 8 Closer Look at NIBIB

**Fig. 6:**
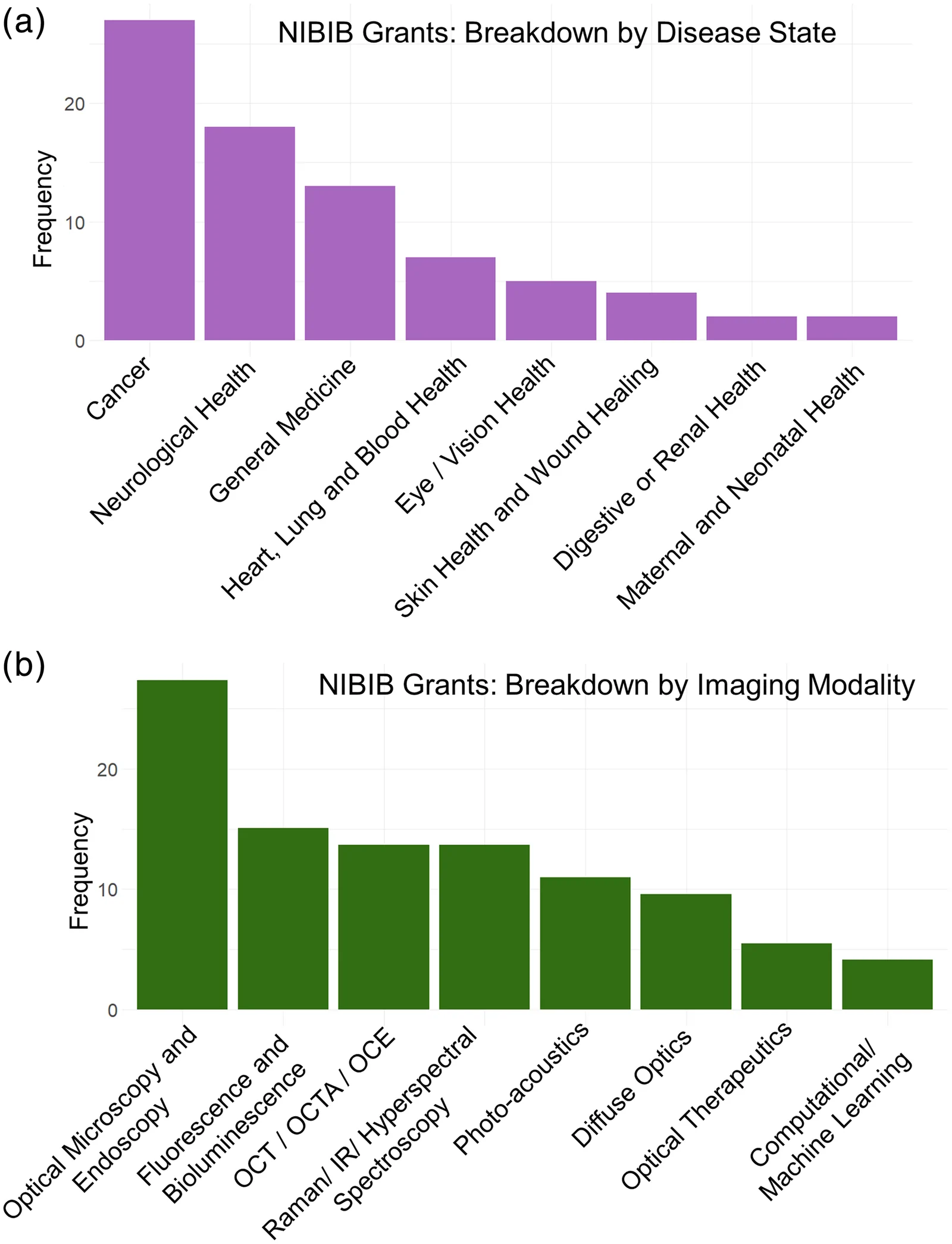
Within the research grants funded by the National Institute of Biomedical Imaging and Bioengineering (NIBIB), panel (a) shows the breakdown of these grants based on the imaging modalities studied, based on the abstract, and panel (b) shows the breakdown of these grants based on the disease states studied, based on the abstract.

Because NIBIB uniquely spans multiple organ systems and disease areas,^14^ we examined its portfolio more closely. Although NIBIB received around 1.1% of the NIH’s total extramural portfolio for FY 2024 (∼$383 million), ∼$29 million of FY 2024 biophotonics funding came from NIBIB, representing 10% of the total investigator-anchored funding. Compared with the overall optical modality distribution, NIBIB invests more heavily in photoacoustics, Raman spectroscopy, hyperspectral and infrared methods, and diffuse optics. These trends reflect NIBIB’s mission to support early-stage and enabling technologies with broad translational potential (Fig. 6).

Biomedical optics work funded by NIBIB remains concentrated in cancer and neurological applications, although its modality spread indicates emerging growth areas where newer optical tools are gaining traction.

## 9 Strategic Implications and a Call to Action

Biophotonics has a long and successful history of advancing cancer, neuroscience, and eye and vision research. These domains have provided natural proving grounds where high-resolution imaging, minimally invasive access, and multiparametric sensing directly support diagnosis and therapeutic guidance. Continued innovation in these well-established areas remains essential, and the community should feel proud of its deep impact.

At the same time, this strong gravitational pull toward traditional applications may constrain our future influence if it prevents engagement with evolving national health priorities. Substantial opportunities exist for optical technologies to transform health outcomes in cardiovascular and metabolic disease, infectious and immune disorders, aging-related decline, maternal and child health, and global health. Current underrepresentation in these domains may reflect multiple factors, including lower submission rates to these institutes, gaps in technology maturity or translational bottlenecks, historical trends in funding, or structural barriers to interdisciplinary collaboration. Given this, progress in these areas will require sustained relationship-building with new clinical partners and programmatic advocacy with institutes that do not yet routinely invest in biophotonics. It will also require leadership willing to accept short-term uncertainties in pursuit of long-term health impact.

Importantly, expanding into underrepresented areas would catalyze new modes of optical innovation. Wearable and home-based optical systems could enable longitudinal monitoring for hypertension and renal disease, directly addressing major drivers of U.S. morbidity. Resource-efficient and battery-powered sensing approaches could extend optical diagnostics to low-resource and rural settings, supporting infectious disease control and maternal health surveillance. These technology directions complement global initiatives around digital health, personalized care, and point-of-need diagnostics, areas of growing NIH investment and policy interest.

There are concrete actions the community can take now. Major conferences can feature dedicated tracks in these emerging clinical domains and actively recruit participation from disease-focused investigators and NIH program leadership. Community journals can organize special sections emphasizing successful clinical exemplars and implementation pathways. Engagement with nontraditional study sections, trans-NIH initiatives, and clinician-researcher networks can broaden both the visibility and the impact of biophotonics.

Ultimately, the future success of our field depends on balancing two forces: sustaining the deep strengths and institutional partnerships developed in cancer, neuroscience, and vision science while intentionally expanding into new health domains where the potential benefit to patients and society is profound. Progress will require shared leadership between the community and NIH, aligning scientific ambition with national priorities. The path forward is not solely a matter of technology readiness; it is also a matter of strategy and the willingness to redefine where optical science can make the greatest difference.

## 10 Limitations

This analysis used a practical but necessarily limited definition of the biophotonics community, based on SPIE databases available to the authors and editorial board members of journals in the field, which may not capture all investigators in the field. Future work could expand this search to include additional databases. Abstract-level LLM analysis may miss important contextual details contained in full applications. Other significant research sponsors, including the National Science Foundation, Department of Defense, and Department of Energy, and major foundations, were not evaluated. Finally, these data provide a snapshot of a single fiscal year. Longitudinal analysis will be important to assess temporal trends, institute shifts, and emerging thematic growth.

## Supplementary Material

10.1117/1.BIOS.3.2.020601.s01

10.1117/1.BIOS.3.2.020601.s02

## Data Availability

The final dataset of investigators, grants, and annotations is available in a spreadsheet as Supplementary Material 1. A summary of prompts and decision rules used in the analysis of the data is provided as Supplementary Material 2. Additional code and data, if required, can be provided upon reasonable request.
