# Supplementary material for: NIH funding in the biophotonics community
Source: Biophotonics Discov. 2026 Jun 5;3(2):020601. doi: 10.1117/1.BIOS.3.2.020601 (PMC13559162; doi:10.1117/1.BIOS.3.2.020601)
Supplement: Supplementary file 1 [file BIOS_003_020601_SD002.docx]

**NIH Funding in the Biophotonics Community**

**Aarohi Mahesh Mehendale**^a^**, Darren Roblyer**^a,b^

a. Boston University, Department of Biomedical Engineering, Boston, MA
b. Boston University, Department of Electrical and Computer Engineering, Boston, MA

**Supplementary Material**

**Summary of prompts to LLM and ChatGPT**

*“Given this excel spreadsheet with project titles and abstracts, come up with several (less than 10) overarching categories that these projects can fit into. Specifically, make these categories related to the specific technology being developed or the imaging/spectroscopy method being studied. For example, Diffuse Optics or Photoacoustics or Image processing/computational are examples of technology categories that should be used. If more than one technique is used, choose a dominant category.”*

Further refinement was performed:

*“I want the number of other/unclassified to be very small, so add categories that make sense. To do this, you need to analyze all the abstracts and see which additional categories make sense to add. I still don’t want too many categories, try to keep it around 10. You can make a non-optical imaging category that would include MRI/CT/PET and related technologies.”*

After these prompts, the user checked random abstracts and their associated categories for accuracy, and further refinement was performed, with the following prompts:

*“You need to go through each title and abstract and look at all the text, not just the first part, and then identify what the optical technique is, and then make sure your categories are correct.”*

After another round of user checking, the following prompt was used to complete the task:

*“Your new classifications are quite good, keep doing this for the remainder of the abstracts.”*

For categorization by disease state:

*“Given the following abstract, what disease state are the researchers focusing on? Answer with the name of one of the following categories: Cancer, Neurological Disorders, Heart/Lung and Blood, General Medicine, Allergies, Eyes, Diabetes/Digestive/Kidney, Skin, Dental, or Other. Make a note if the abstract has to do with Maternal or Neonatal Health, or has relevance to resource limited settings. The abstract is:”*

*“Given the following abstract, does the project focus on applications for women’s, maternal, or neonatal health? Read the abstract carefully to determine this, do not use a simple word-matching algorithm. Answer only with 'yes' or 'no'. The abstract is:”*
